# Blood Lead Level Elevation as a Result of a Retained Bullet

**Published:** 2015-01-01

**Authors:** Sevgi Buyukbese Sarsu, Mehmet Akif Buyukbese

**Affiliations:** 1 Gaziantep Children's Hospital, 27060, Gaziantep, Turkey.; 2 MD, Professor of Internal Medicine, Atakoy Dünyagöz Hospital, Istanbul, Turkey.

**Dear Sir,**

Increased level of lead after gunshot injury is very rare.[1] Higher serum levels frequently develop after the ingestion of dye containing lead, dust and soil. Lead may be toxic at all ages but children are affected more than adults. Herein we report such an incident.

A 6.5-year old girl was brought to the emergency room with a gunshot injury. Bullet entry hole was under the right scapula, but no exit wound was found. Abdominal examination revealed no tenderness. No free air was seen under the diaphragm on the plain radiogram. Abdominal CT scan demonstrated minimal contusion in the liver and the bullet was detected in the pelvis near the right side of the urinary bladder (Fig.1). Patient was hemodynamically and neurologically stable. Before discharge on the 4th day of the gunshot injury, serum lead level was 1.9µgr/dl. Monthly follow-up was done via radiological evaluation and serum lead levels. At 4 months follow-up, the patient developed mild apathy and irritability. Her serum lead level was found to be 18.0µgr/dl, so a decision for surgical intervention was taken. A single-port trans-umbilical laparoscopy revealed bullet wrapped by the omentum. The bullet was extracted after partial omentectomy. Blood lead levels returned to normal on follow-up with improvement of apathy.

**Figure:**
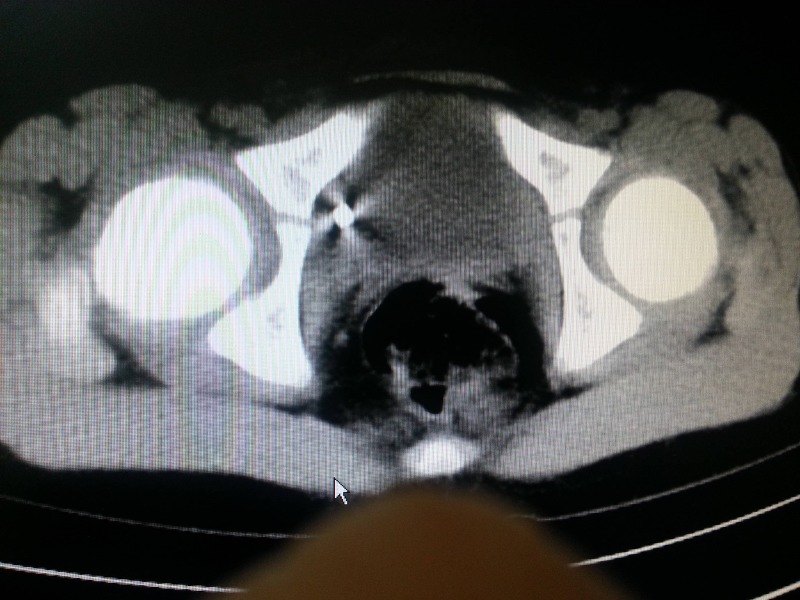
Figure 1:CT scan showing bullet in the pelvis near the right side of the urinary bladder.

Systemic symptoms such as abdominal pain, vomiting, headache, joint pain, loss of appetite, increased blood pressure and irritability may all be associated with increased lead levels.[2] In our case non-specific mild irritability and apathy were observed. Bullet fragmentations may contribute to lead absorption.[3] Correlation of bullet location and lead toxicity is also described. A bullet in proximity to the body fluids (cerebrospinal, pleural, synovial space etc.) may lead to increase in the absorption rate and thus elevated blood lead levels.[3]

In our case, the bullet was entrapped by the omentum which has very rich blood supply that might have contributed to lead absorption. Usually gunshot abdominal injuries are managed surgically however in selected case like ours, conservative approach may be applied. In all such children with retained bullet, a careful follow-up with monthly lead levels should be mandatory to pick the cases up before lead toxicity establishes. Gradual elevation of blood lead levels necessitate operative removal of the retained bullet.

## Footnotes

**Source of Support:** Nil

**Conflict of Interest:** None declared

